# Seasonal plasticity in daily timing of flight activity in *Anopheles stephensi* is driven by temperature modulation of dawn entrainment

**DOI:** 10.1098/rstb.2023.0343

**Published:** 2025-01-23

**Authors:** Samuel S. C. Rund, Aidan J. O'Donnell, Kimberley F. Prior, Daan R. van der Veen

**Affiliations:** ^1^ Department of Biological Sciences, Center for Research Computing, and Eck Institute of Global Health, University of Notre Dame, Notre Dame, IN 46556, USA; ^2^ Institute of Ecology and Evolution, School of Biological Sciences, University of Edinburgh, Edinburgh EH9 3FL, UK; ^3^ Chronobiology Section, Faculty of Health and Medical Sciences, University of Surrey, Guildford GU2 7XH, UK

**Keywords:** circadian, temperature entrainment, mosquito, malaria, invasive species, light entrainment

## Abstract

The Asian malaria vector *Anopheles stephensi* is invading Africa, requiring it to adapt to novel climates and ecosystems. In part, this may be facilitated by *An. stephensi*’s poorly understood seasonal behavioural plasticity in flight timing, leading to earlier biting activity in cold Asian winters and later biting times in the warm summer. Changes in behavioural timing could be directly imposed by seasonal variation in ambient light and temperature levels or result from altered entrainment of intrinsically expressed circadian rhythms by these factors. We demonstrate that *An. stephensi* entrained flight activity timing is phase-locked to dawn and is not affected by constant ambient temperature, which cannot explain earlier biting activity in colder winters with later dawn. Instead, we show that where night temperatures are the colder part of daily temperature cycle; the entrained phase-angle between dawn and flight activity is altered, hereby increasingly colder, winter-like nights progressively advance flight activity onset. We propose that seasonal timing plasticity optimizes behaviour to warmer daytime in winter, and colder nights in summer, providing protection against both heat-desiccation and cold immobility. The adaptive advantage of this plasticity could be relevant to the successful invasion and survival of *An. stephensi* in African climates, and changing climate worldwide.

This article is part of the Theo Murphy meeting issue, ‘Circadian rhythms in infection and immunity’.

## 1. Introduction

*Anopheles stephensi* is a sub-tropical southern-Asian [1–3] insect vector of *Plasmodium* malaria parasites, and is now also a highly concerning invasive vector species to urban Africa [4–7]. Recent estimates suggest that *An. stephensi*’s invasion of Africa may put an additional 126 million people at risk of malaria [8]. In many vector species (e.g. *Aedes caspius*, *Aedes detritus*, *Culex pipiens* and *Culicoides sonorensis* [9,10]), the time of day that biting (and thus likely pathogen transmission) occurs is reported to vary by season. This seasonal variability in the timing of flight activity is an example of phenotypic plasticity, which occurs when different phenotypes are observed from the same genotype under different environmental conditions [11]. A similar seasonal plasticity in activity timing to that seen in these mosquitoes has also been observed in the Asian population of *An. stephensi*, with earlier evening biting times in winter [2,12]. Given that this seasonal plasticity carries the adaptive benefit of protecting against both heat desiccation and immobility during cold, it may also facilitate the invasion of Africa, which has very different ecosystems. However, we currently lack even a basic understanding of the biting times of the African populations of *An. stephensi*, owing to the rarity of field collections and observations of adult *An. stephensi* mosquitoes in this area [4,6,13–15]. Understanding the drivers of seasonal plasticity in biting times is highly relevant to the efficacy of bed nets (which relies on the alignment of daily timing of human and mosquito behaviours [16]) and also helps in determining whether this plasticity is an adaptive advantage facilitating *An. stephensi*’s invasion of Africa.

Many studies, both in the lab and in wild settings, have offered evidence that seasonal changes in insect behaviours (such as flight activity) may be occurring in direct response to environmental light and temperature levels (known as ‘masking’ [17]). Examples of such masking in mosquito behaviour include male *Anopheles superpictus* mosquitoes which can be induced to form a (normally dusk-mediated) mating swarm in the laboratory by simply reducing environmental light intensity during the day [18]. *Anopheles gambiae* mosquitoes are also proposed to time their onset of nocturnal activity as a visual response to dusk [19], and *Culex coronator* mosquitoes have an approximately 10 min advanced (earlier) peak in biting in forest populations than mosquitoes that were found living in brighter conditions outside the forest [20]. These studies show the immediate and masked responses to light in keeping activity in the same phase relationship to dusk, but cannot explain the behavioural plasticity observed in *An. stephensi*, which changes its phase relationship to dusk between the seasons, such that in winter activity onset takes place before dusk, and in summer activity onset takes place after dusk [2,12].

Besides the immediate and masked effects of light on timing of mosquito activity, the daily timing of behavioural activity has also been shown to be driven by an internal (endogenous) biological circadian (24 h) timing system that evolved several times independently [21]. Circadian clocks organize at different neuronal or tissue levels [22–25] to drive behavioural and physiological rhythms such as locomotion, detoxification and host-seeking behaviours in mosquitoes [26–28]. The circadian timing system is synchronized (entrained) to external environment cues, known as Zeitgebers, of which light is often seen as the strongest cue [29]. Given that the seasonal changes in flight activity of *An. stephensi* are unlikely to be caused by the masking effects of light, then it is possible this light entrainment of intrinsic timing may vary between seasons owing to changes in non-photic environmental conditions, such as temperature or humidity. The non-photic conditions could directly mask intrinsically timed activity; however, cycles in non-photic cues such as temperature are also frequently reported to serve as zeitgebers that entrain daily timing [22,23,30–35].

While non-photic entrainment has been demonstrated in the mosquito *Culex pipiens pallens* [36]*,* it has not yet been demonstrated in *An. stephensi*. Indeed, mosquitoes have receptors that can directly detect heat and humidity, which are utilized for host-seeking and for finding egg-laying sites [37] but have not yet been implicated in daily timing or circadian entrainment. These non-photic sensing pathways could modulate a seasonal phenotype by direct masking of light-entrained activity, could drive thermosensitive changes in the circadian clock [38], or may serve as non-photic Zeitgebers themselves [34]. To date, however, it remains unknown whether *An. stephensi* entrains to temperature or humidity, and whether these factors are involved in driving seasonal changes in diurnal timing of flight activity.

In this paper, we set out to investigate these potential masking and entraining effects of temperature and humidity on light–dark entrainment of behaviour in *An. stephensi*, with a view to characterize how changes in the timing of these cues can drive seasonal plasticity in biting times. Understanding the seasonal entrainment of malaria vectors, and being able to predict their peak activity across the year, may allow determination of the efficacy of bed nets (which is critical for malaria control operational planning [39]) as well as underpin novel prevention strategies such as time-of-day-optimized adulticidal insecticide fogging. Besides the direct relevance to biting prevention strategies, understanding what drives the plasticity in entrainment is also key to the larger questions around how the Asian *An. stephensi* vector could invade and survive in hot African climates.

## 2. Results

Overall behavioural flight activity of female mosquitoes that were given the opportunity to mate was recorded during a 12 h: 12 h light–dark (LD 12:12) cycle by placing individual mosquitoes in glass tubes inside a locomotor activity monitor (LAM) unit [19]. Mosquitoes exhibited a 7.62 h between-individual variation in onsets of flight activity, ranging from early- to mid-dark phase (Zeitgeber time (ZT) 13.68 to ZT 21.3, electronic supplementary material, figure 1A), with a mean nightly activity onset at ZT 16.70 ± 0.14 (*n* = 97), mean ± SEM, ZT 0 is defined as lights on). We investigated whether this variability in timing of flight onset in entrained conditions was associated with intrinsic circadian period (where short intrinsic period could drive an earlier phase of entrainment [40]) by releasing the mosquitoes in constant darkness after entrainment. Mean free-running period was 24.5 ± 0.11 h (mean ± SEM) and exhibited low between-individual variation that did not associate with timing of individual flight onset in LD 12:12 (linear regression, *R*^2^ < 0.01; electronic supplementary material, figure 1B). Given the large inter-individual variation in onset of flight activity, we used within-individual shift in timing of onsets of flight activity (Δ onset) to quantify changes in entrainment of flight activity in further experiments.

### (a) *Anopheles stephensi* flight activity timing is entrained to dawn

**Figure 1.**
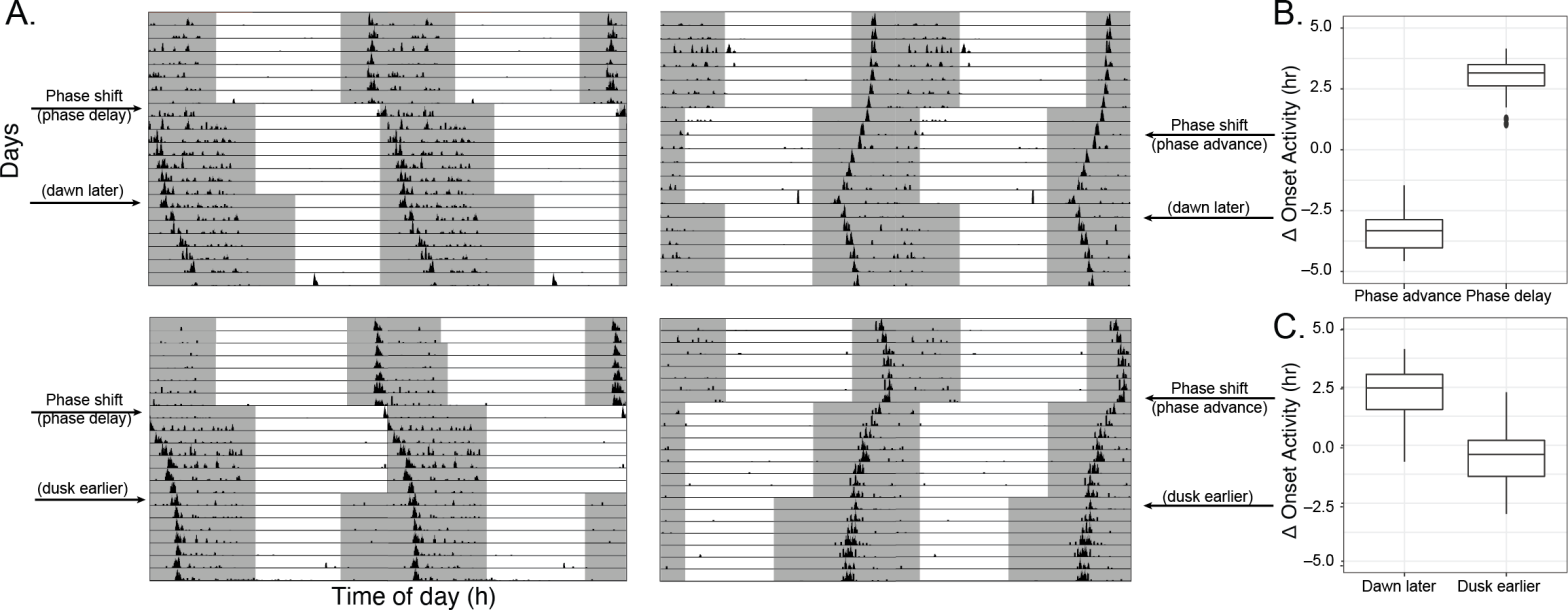
*Anopheles stephensi* mosquito flight activity entrains to dawn, and not dusk. (*a*) Actograms of flight activity in black, with darkness (lights off) indicated in grey, and lights on as white background. Mosquitoes were entrained to a 12 h : 12 h light–dark photocycle, 60% RH, 26°C, with 1 h dawn and dusk transitions and activity monitored for 7 days. Mosquitoes were then either phase-delayed or -advanced (left and right panels respectively) and activity monitored for a further 7 days. Finally, the photoperiod was compressed, resulting in an 8 h : 16 h light–dark photocycle, by shifting either dawn or dusk by 4 h, and mosquitoes were again monitored for 7 days. A representative actogram of a mosquito in each of the four protocols is shown. (Data are double-plotted such that each cycle is duplicated, for visualization purposes). (*b*) Phase shift in onset of activity (clock hours) following a phase delay (dawn/dusk cycle 4 h later) or phase advance (dawn/dusk cycle 4 h earlier) of each individual mosquito. (*c*) Phase shift in onset of activity (clock hours) following a 4 h photoperiod compression (by delaying dawn or advancing dusk of each individual mosquito).

To establish whether seasonal differences in onset of nightly activity occurred in response to seasonal differences in the external light–dark (LD) cycles, we next looked at light entrainment of *An. stephensi* flight activity. Four groups of mosquitoes were entrained (*n* = 32 per group) to LD 12:12 cycles for 7 days, followed by either a 4 h phase advance (2 groups) or 4 h phase delay (2 groups) in the light–dark cycle, subsequently followed with an extension of the dark period to 16 h by either delaying dawn or advancing dusk (see figure 1*a* for experimental design). All mosquitoes shifted timing of their flight onset in response to a 4 h phase shift in the light–dark cycle, resulting in a mean within-individual shift on the seventh day of 3.07 ± 0.14 h (mean ± SEM, paired *t*‐test, *t*(−6.9) = 45, *p* < 0.001) in the delay groups, and −3.39 ± 0.11 h (mean ± SEM, paired *t*‐test, *t*(30.4) = 50, *p* < 0.001) in the advance groups (unpaired *t*‐test, advance versus delay, *t*(−41.262) = 94.903, *p* < 0.001; see figure 1*b*).

Strikingly, in the dark extension protocol, we found that mosquitoes shifted flight onset timing solely in response to a delayed dawn (2.36 ± 0.17 h, mean ± SEM, paired *t*‐test *t*(−3.7) = 47, *p* < 0.001) (top panels, figure 1*a*), but not to an advanced dusk (−0.61 ± 0.15, mean ± SEM, paired *t*‐test *t*(−1.7) = 48, *p* = 0.09; figure 1*c*, and bottom panels, figure 1*a*). Overall, this shows that *An. stephensi* entrains to a light–dark cycle, where dawn timing, but not dusk timing, is the critical timing cue (Zeitgeber). In this protocol, the shift in the onset timing is less than or proportionate to the shift in the light–dark cycle, and onset of activity moves into the night during winter-like long nights, which is opposite to the seasonal phenotype seen in the field.

### (b) *Anopheles stephensi* flight activity timing is not affected by changes in ambient temperature or humidity

**Figure 2.**
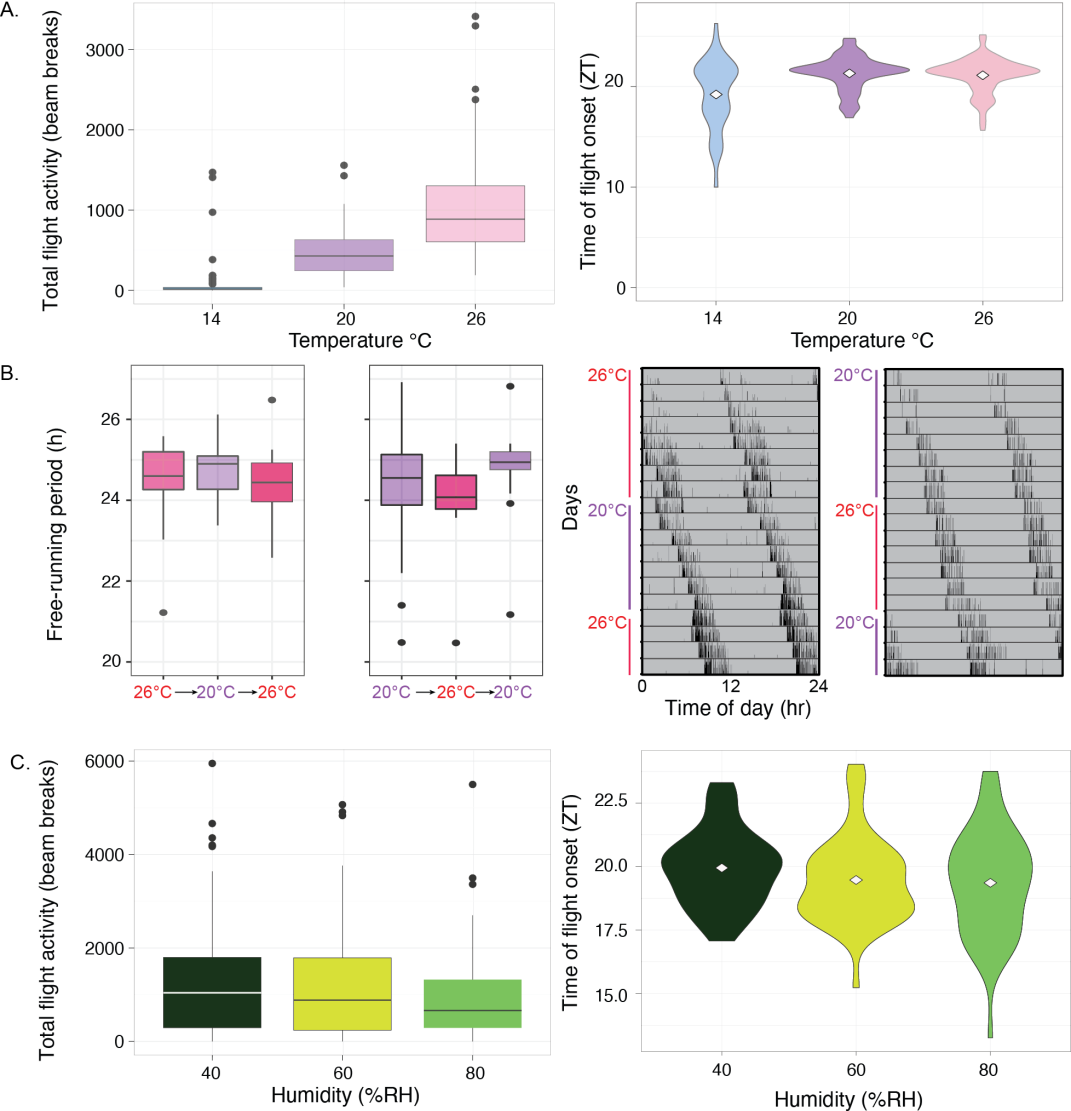
Effect of ambient temperature and humidity on total flight activity, onset timing and free-running period. (*a*) Mosquitoes were placed in one of three starting conditions; 14, 20 or 26°C ambient temperature. After 3 days, the conditions were adjusted to a different ambient temperature, and again after another 3 days, ensuring that all mosquitoes were exposed to all three temperatures successively, but in differing orders. Boxplots show total flight activity (left) and violin plots the times of flight onset (right). There was a significant difference by temperature in the amount of daily activity, but not the time of onset of activity. (*b*) Same approach as in (*a*), but now in constant darkness. Mosquitoes were placed in one of two starting conditions; 20 or 26°C ambient temperature. After approximately 7 days, the conditions were adjusted to the other ambient temperature, and again after another 3 days they were returned to their original temperature. No pronounced change in free-running period was noted. Only animals that survived the entire assay regime were included (*n* = 50). (*c*) In an identical experimental design to flight activity at different temperatures, but investigating alternative humidity levels of 40, 60 and 80% RH; there was a significant difference by humidity level in the amount of daily activity, but not the time of onset of activity.

To establish whether a seasonal circadian phenotype could be linked to changes in light–dark entrainment mediated by ambient temperature or relative humidity levels, we entrained mosquitoes (*n* = 96) to LD 12:12, and sequentially exposed them to three ambient temperatures (14, 20 and 26°C) for 7 days for each temperature in differing, sequential orders (*n* = 31 or 32 mosquitoes per condition; electronic supplementary material, figure S2) at a constant 60% RH. As expected for ectotherms, increasing ambient temperature led to a pronounced increase in total activity per day (one-way repeated measured ANOVA, *F*_2,186_ = 141.8, *p* < 0.001). We observed mean total activity of only 72 ± 23 beam breaks per day (± SEM) at 14°C, 479 ± 31 at 20°C, and 1023 ± 64 at 26°C (figure 2*a*; electronic supplementary material, figure S2.)

Across the different ambient temperatures, mean onset time was ZT 20.55 ± 0.27 (mean ± SEM), and onset of flight timing occurred at ZT 19.21 ± 0.35 at 14°C, ZT 21.12 ± 0.18 at 20°C, and ZT 21.31 ± 0.018 at 26°C (mean ± SEM; figure 2*b*). The effect of ambient temperature on onset time was found to be significant (one-way repeated measure ANOVA, *F*_2,186_ = 21.7, *p* < 0.001) with regards to 14°C compared with both 20 and 26°C (*post hoc* tests with Bonferroni correction). However, owing to the extremely low activity level of the mosquitoes at 14°C, making activity onset difficult to determine and leading to a substantial range of onset timing estimates (figure 2*a*; electronic supplementary material, figure S2), we interpreted this finding at 14°C as unreliable.

A key characteristic of circadian clocks is that they are temperature-compensated [41]; so we housed another group of mosquitoes under the same temperature conditions as before, but now in constant darkness, to determine intrinsic period (*n* = 50 per group). Behavioural activity at 14**°**C was insufficient to determine an intrinsic period, but intrinsic periods did not differ between 20**°**C (24.67 ± 0.12 h) and at 26**°**C (24.34 ± 0.1 h; figure 2*b*; one-way repeated measured ANOVA, *F*_5,116_ = 2.19, *p* = 0.06). As expected, this demonstrates that mosquito circadian clocks are temperature-compensated, and that a seasonal phenotype could not be caused by temperature-mediated changes to intrinsic period.

We next investigated the effect of ambient relative humidity on timing of flight onset by exposing LD 12:12 entrained mosquitoes to three 7 day stretches of constant relative humidity of 40, 60 and 80% in differing, sequential orders at a constant 26°C temperature (*n* = 12–22 mosquitoes per condition). We observed total activity of 1611 ± 165 (mean beam breaks per day ± SEM) at 40%, 1496 ± 178 at 60%, and 1180 ± 107.3 at 80% RH, which was low-magnitude, but significantly different (one-way repeated measured ANOVA, *F*_2,96_ = 21.7, *p* = 0.041; figure 2*c*). We did not find any difference in timing of nightly flight onset, which was ZT 19.95 ± 0.21 at 40%, ZT 19.47 ± 0.25 at 60%, and ZT 19.35 ± 0.30 at 80% RH (mean ± SEM, one-way repeated measure ANOVA, *F*_2,96_ = 3.0, *p* > 0.054), with an overall mean onset of ZT 19.59 ± 0.26 (mean ± SEM) (figure 2*c*).

### (c) Thermocycles alter phase of entrainment

In Pakistan and India, the natural Asian habitat of *An. stephensi*, summer and winter differ in mean day and night temperatures and duration [2]. We thus next investigated whether daily cycles of temperature (thermocycles) could entrain circadian rhythms in flight onset and alter light–dark entertainment. Because *An. stephensi* overall activity is very low at winter temperatures, and is an insufficient activity for analysis, we first focused on duration of day and night variation of temperatures, using only summer-like temperatures.

**Figure 3.**
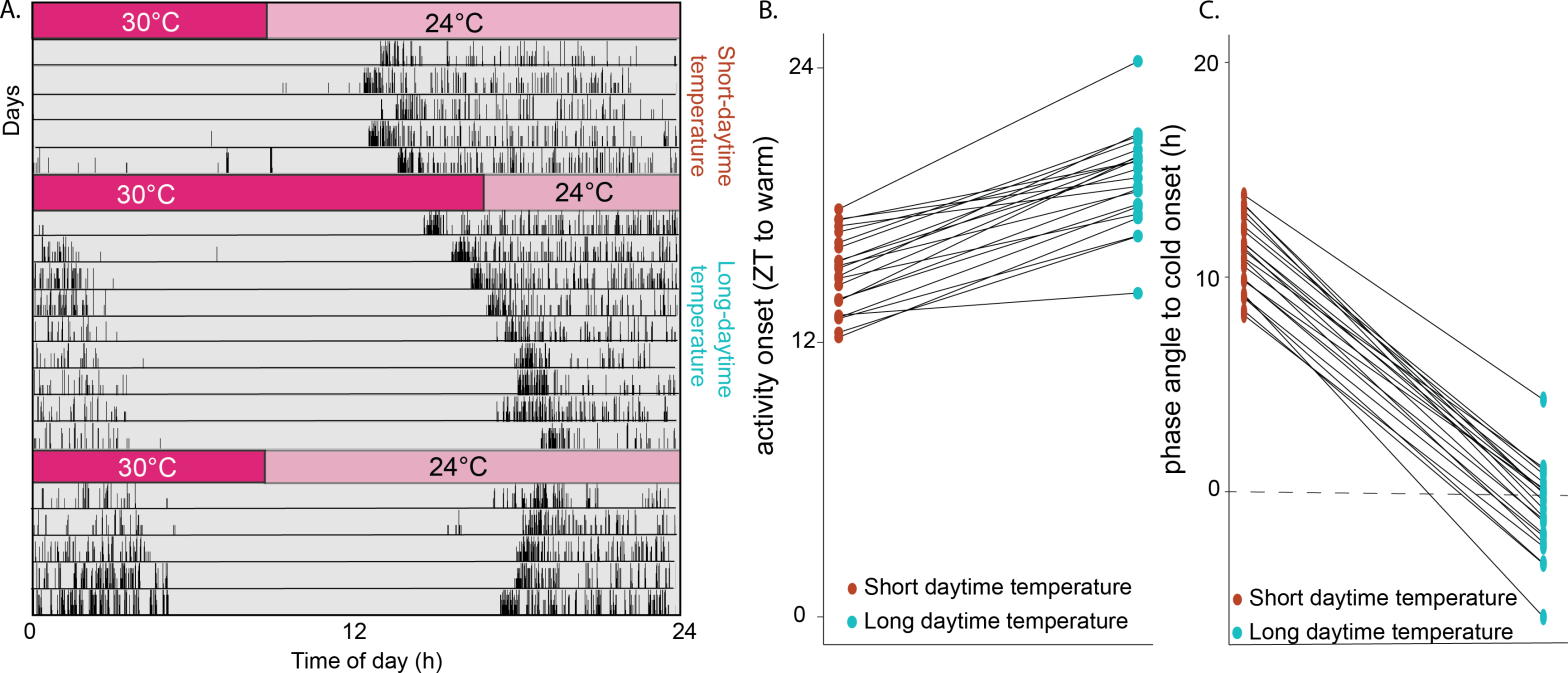
Mosquitoes entrain to thermocycles. (*a*) Representative actogram of mosquitoes held in constant darkness, entrained to a summer-like (long-daytime temperature) and winter-like (short-daytime temperature) thermocycle. Under dark–dark conditions, mosquitoes were entrained to a summer-like temperature range (warm = 30°C, cold = 24°C) with an 8 h : 16 h warm : cold thermocycle, then conditions were changed to a 16 h : 8 h warm : cold thermocycle, and then back to the original 8 h : 16 h warm : cold thermocycle. (*b*) Plot indicating the within-individual shift in onset time, between long and short thermoperiods (left), and phase difference between onset of the cold period and activity onset (right) under long and short thermoperiods.

In constant darkness, mosquitoes were exposed to winter-like short daytime temperatures (30°C, 8 h) and long nighttime temperatures (24°C, 16 h). We then changed the thermocycle to a summer-like 16 h day and 8 h night with temperatures of 30 and 24°C respectively, and reverted to the original thermocycle after 9 days, when stable entrainment was achieved (figure 3*a*; *n* = 22). Under winter-like, short-daytime temperature, flight onset occurred 15.02 ± 0.36 h (mean ± SEM) after the start of daytime temperature, which was delayed to 19.14 ± 0.44 h (mean ± SEM) after the start of daytime temperature when switching to the summer-like longer daytime temperature (figure 3*b*). As a result, the phase angle between the start of nighttime temperature and the onset of flight activity reduced from 11.14 ± 0.45 h (mean ± SEM) in the winter-like, long night compared with the summer-like, short night to −0.98 ± 0.37 h (mean ± SEM, paired *t*‐test, *t*(21) = 12.025, *p* < 0.001; figure 3*c*). When returned to the long winter-like nights, activity onset began to re-entrain to an earlier phase; but owing to lifetime constraints under these conditions, the experiment ended before stable entrainment was achieved.

### (d) A combination of photocycle and thermocycle drive seasonal phenotype in the laboratory

**Figure 4.**
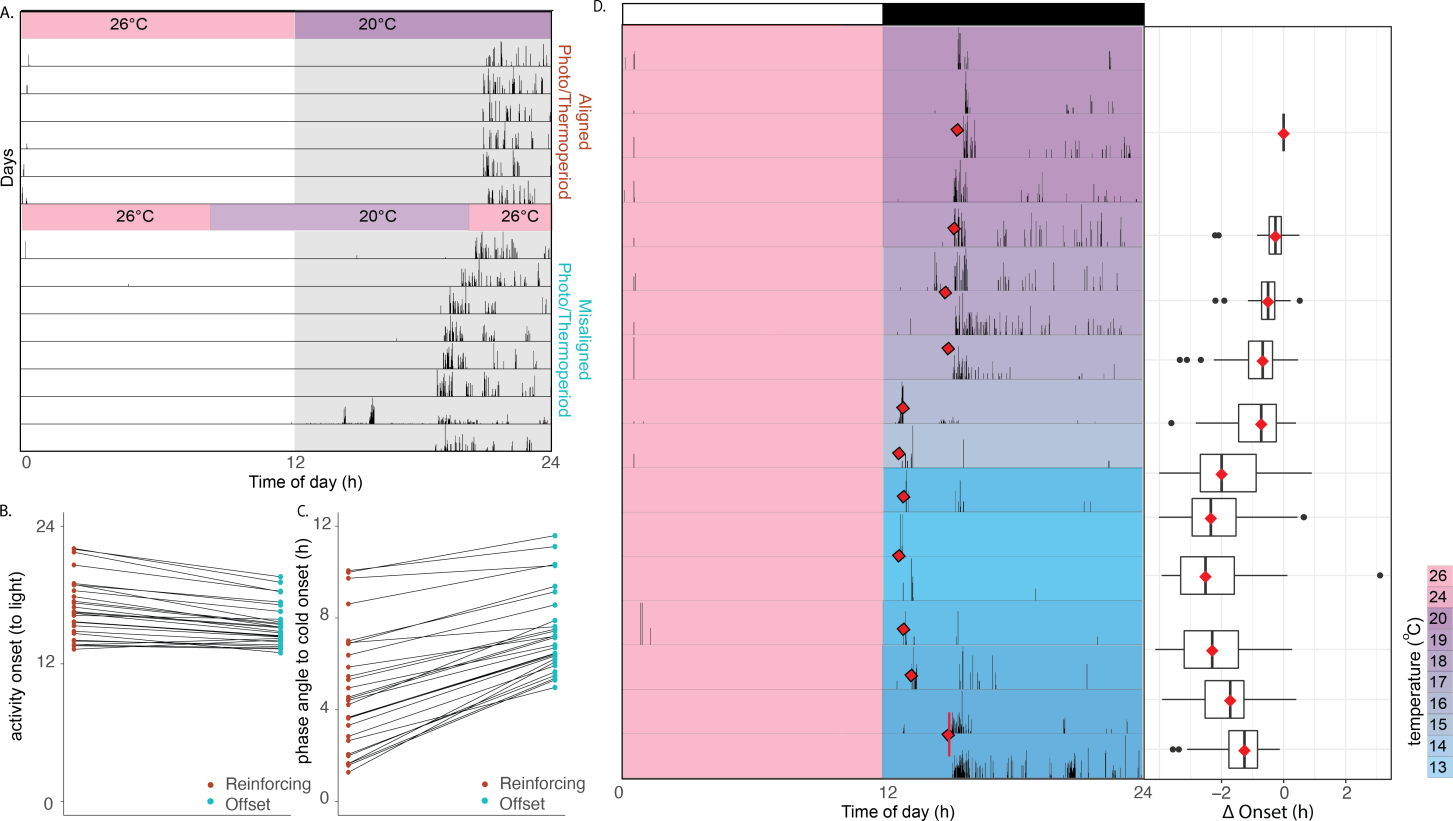
A combination of photocycle and thermocycle drive seasonal phenotypes. (*a*) Representative actogram of mosquitoes exposed to a reinforcing thermocycle followed by a 4 h offset of thermocycle from photocycle. Note the presence of transients. (*b*) Plot indicating the within-individual shift in onset time, between reinforcing thermocycles. (*c*) Phase difference between onset of the cold period and activity onset under reinforcing and offset thermocycles. (*d*) The relationships between ambient temperature and the thermocycle with onset time. Ambient temperature changes the entrained phase relationship between dawn and behavioural activity onset. Mosquitoes were entrained for several days to a 12 h : 12 h light–dark photoperiod with a daily reinforcing 12 h : 12 h 26°C : 20°C thermoperiod. The thermocycle was modified such that the daytime temperature always stayed at 26°C, but descended to lower and lower nighttime temperatures on subsequent nights. As the evening temperature gets colder, the onset of activity became earlier relative to the light–dark cycle. This is reversed as the nadir of the thermocycle gets warmer. A representative actogram is shown as well as a graph demonstrating the change in onset normalized to the starting condition per animal (as onset times vary so dramatically between animals; see electronic supplementary material, figure S1).

The delayed onset of flight activity relative to the start of daytime temperatures in summer-like conditions mirrors what was reported in *An. stephensi* in the wild [2], and is contrary to the advance seen under summer-like light–dark cycle conditions. To address this paradox between light and temperature entrainment, we next explored how temperature entrainment interacts with light–dark entrainment to drive a seasonal phenotype. Using an experimental approach that does not recapitulate any natural condition, we entrained mosquitoes (*n* = 28) to an LD 12 : 12 cycle accompanied by an aligned 12 h : 12 h thermocycle (26 : 20°C), and then advanced the thermocycle by 4 h, while keeping the light–dark cycle unchanged (figure 4*a*). The resulting change in timing of flight onset was an advance (relative to the light–dark cycle) of 1.44 ± 0.20 h (mean ± SEM; figure 4*b*, left panel). After the 4 h advance in the thermocycle (relative to the light–dark cycle) the phase angle between the start of nighttime temperature and the onset of flight activity increases from 4.81 ± 0.50 h (mean ± SEM) to 7.37 ± 0.34 h (mean ± SEM, paired *t*‐test, *t*(27) = −12.894, *p* < 0.001; figure 4*b*,c). This shows that the new time of the onset is intermediate to the one driven entirely by light or driven entirely by temperature cycles, thus demonstrating the timing of mosquito onset is entrained by a combination of temperature and light cycles.

With the understanding that the photo- and thermocycle interact to entrain behavioural timing, we next set out to expose how the seasonal phenotype observed in the field can be explained by this interaction. Mosquitoes (*n* = 32) were entrained for several days to 12 : 12 LD photoperiod with a daily 12 h : 12 h thermoperiod with a 6°C amplitude (26°C : 20°C day : night). The cold-phase temperature of the thermocycle amplitude was then decreased by 1°C increments over several days, from 20 to 13°C, before this was reversed and the temperature was incrementally raised back up to 16°C. This was accomplished by always keeping the daytime temperature at 26°C but changing the nighttime temperatures. As the evening temperature got colder, the onset of activity became progressively earlier relative to the light–dark cycle. This was reversed as the nighttime temperature of the thermocycle got warmer again (figure 4*d*).

## 3. Discussion

**Figure 5.**
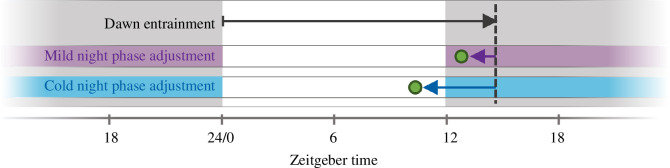
Schematic representation of the plastic phasing of onset of flight activity (green dot) in mild and cold nights in *Anopheles stephensi*. Dawn entrainment sets a stable nighttime phase (black line) in the light–dark cycle (grey nights), and the timing of flight activity onset is proportionally advanced in milder (purple) or colder (blue) nights.

In Asia, *An. stephensi* displays seasonal variations in biting time, with earlier biting in the winter months than in the summer months [2,12]. The environmental cues that drive this seasonal variation are not known and questions remain around the ability of *An. stephensi* to invade Africa from Asia and thrive in different climates. Here we describe that, in the laboratory, the daily activity patterns of *An. stephensi* are synchronized to dawn, not dusk. Such a dawn-only synchronization seemingly limits the mosquito’s ability to sense seasonal changes in photoperiod, or advance its flight activity to the warmer day in winter as seen in the field. We show that progressively lowering nighttime temperatures in daily temperature cycles associates with increasingly earlier biting times, but that this is not a response to ambient temperature *per se* and must occur in temperature cycles. We propose that in the field, lower nighttime temperature in winter advances the time of biting the next day in a proportional way—meaning that a mild winter night associates with less of an advanced biting time the next day than a colder winter night (figure 5). These findings describe a seasonal plasticity driven by combining dawn timing and nighttime temperature in thermocycle entrainment, which is highly plastic and of interest given the poorly understood ability of this Asian malaria vector to invade and thrive in hot and arid environments of Africa.

We observe that timing of onset of flight activity in *An. stephensi* is shifted in response to changes in dawn timing, but not dusk timing, suggesting dawn is the principal timing cue (Zeitgeber) in light–dark entrainment for this species. Crucially, this suggests that daylength (photoperiod) does not drive a seasonal change in entrainment of flight onset, although this was not directly tested in this paper. Photoperiod is often reported to be a seasonal indicator for insects [42], including *Anopheles*, although some other exceptions have been reported [43,44]. Many mosquito species are known to sense season through daylength, which sometimes induces a winter diapause which can result in dramatic behavioural and physiological changes [45]. Interestingly, diapause and aestivation (prolonged torpor) have been reported in several anophelines [46,47], but to our knowledge neither phenomenon has been described in *An. stephensi*. Dawn entrainment and absence of a photoperiod response lead to a delayed onset of flight activity in winter-like light–dark cycles in the laboratory, which starkly contrasts to the observed earlier winter flight times in the field [2,12]. As a terrestrial ectotherm, the winter advance in diurnal timing of biting activity reported in wild *An. stephensi* would be consistent with optimization towards mid-range temperatures, which lower the risk of desiccation (a significant concern to terrestrial insects in high temperature) but are still warm enough for efficient flying [48–50]. The observed delay in flight timing seen in response to a winter-like photoperiod alone in our experiments does not reflect what is seen in the field and would be maladaptive, suggesting that other seasonal cues may be important to *An. stephensi*.

We show that changes in ambient temperature or humidity do not mask or alter the light-entrained timing of flight onset. This contrasts with findings in other insects, such as *Chironomus yoshimatsui*, in which ambient temperature can shift the activity onset to occur before or after dusk [51], and *Nicrophorus quadripunctatus* and *Culex pipiens molestus*, in which colder ambient temperatures can shift morning peaks to occur later and evening activity peaks to occur earlier [52,53]. However, we do see that *An. stephensi* flight activity is entrained by temperature cycles. Here we demonstrate a reduction in nighttime temperature within this temperature cycle is enough to advance the light-entrained flight onset in an exposure-dependent manner, whereby colder nighttime temperatures advance activity more than milder temperatures. From our findings, it remains unclear whether this change in behavioural timing results from an adaptive change to the phase relationship between activity and the circadian clock, or whether the circadian clock itself is shifted in a temperature-dependent manner. It may also be that ambient temperature alters the light–dark entrainment pathway such that dusk entrainment becomes more relevant in winter-like thermocycles. Although in general circadian clocks are temperature-compensated (i.e. resistant to temperature changes [41]), a thermosensitive splicing event in the 3′ untranslated region of *Period* mRNA has been reported in *Drosophila melanogaster* and attributed to earlier timing of activity in the winter [38]. However, the fact that we only see our phenotype when exposed to temperature cycles (and not under dramatic changes to ambient temperature) suggests a mechanism separate from temperature-dependent splice variants is likely to be involved here and may include a temperature-cycle entrainment mechanism.

We sequentially adjusted nighttime temperature every night in our protocol, which was met with an accompanying shift in flight onset time on the very next day. Biting time of *An. stephensi* in Africa is poorly understood [4,6,13–15], but studies in *An. gambiae* suggest that flight behaviour can be used as a proxy for host-seeking flight behaviour, which in turn reflects biting propensity and potential malaria transmission [51]. While this needs to be further confirmed, the rapid responsive plasticity we see in our protocols suggests that nighttime temperature can be used to predict flight activity the next day. Although the large between-individual variability in onset timing takes away somewhat from the value of this prediction in the field, our findings do suggest that on days with colder nights, mosquitoes may bite prior to the protective hours of bed net use the next day. In very hot regions, there is a risk that mosquito activity could be pulled into the early morning when humans have risen and are no longer protected under bed nets, which would have serious malaria control implications. We acknowledge that our protocol, by lowering only the nighttime temperature, simultaneously increased the amplitude of the temperature cycle. Further investigations will be required to disentangle the effects of these two changes.

The highly plastic ability of *An. stephensi* to shift its activity in response to nighttime temperature we report here may be an adaptive benefit that is facilitating the mosquito's invasion of new ecosystems. Some of the regions where this invasive species has been found are hotter and more arid than where our Asian experimental colony was collected in Lahore, Pakistan. For example, in Khartoum, Sudan, during the hottest months, nighttime temperatures can exceed 33°C and daytime temperatures, 42°C. The ability of *An. stephensi* to invade and survive climates with such temperatures is often attributed to its utilizing man-made containers as larval sources [54] where few natural water sources are available. Our data suggest that *An. stephensi* may have a second adaptation allowing it to invade these arid environments: its ability to avoid high heat and desiccation through plastically shifting its behavioural period to the later, cooler part of the night.

Indeed, our findings pose more general questions around how different mosquito strategies of achieving seasonal plasticity can cope with climate change, and/or facilitate the invasion of ecosystems where the photoperiod is a poor predictor of temperature conditions (such as for equatorial species), or where the temperature and light–dark cycles are aligned differently from in the original habitat. In the case of *An. stephensi,* we believe that it is seasonal plasticity driven by combining dawn timing and nighttime temperature in thermocycle entrainment, rather than responding to photoperiod or ambient temperature, that may have given this mosquito an adaptive advantage. This should be further defined to understand how this Asian malaria vector is able to invade Africa and potentially thrive despite continuing climate change.

## 4. Material and methods

### (a) Mosquito colony and breeding

A laboratory colony of *An. stephensi* (SD500; colonized from Pakistan) was reared at 26°C and 70% RH under a 12 h : 12 h light–dark (LD 12:12) photoperiod, with *ad libitum* access to 8% fructose solution post-emergence.

### (b) Behaviour recording

Individual mosquito locomotor/flight activity was measured with a locomotor activity monitor 25 (LAM 25) system (TriKinetics, Waltham, MA). Individual mosquitoes were placed in 25 mm diameter × 150 mm length clear glass tubes with access to 8% fructose solution in the tubes provided *ad libitum*. Flight activity was quantified as number of infrared beam breaks per minute. Each LAM unit allowed simultaneous monitoring of 32 mosquitoes. While each replicate behavioural recording started with approximately equal numbers of mosquitoes, differing amounts of mortality during the experiments sometimes resulted in an uneven final number of individuals in each group, and all reported group size numbers reflect the number of surviving mosquitoes. See [19] for further details on experimental setup. For the humidity-changing experiment, the tube was modified slightly, with the cotton plug replaced with mesh netting to allow more exposure to the ambient humidity of the incubator.

Environmental conditions within the incubators were monitored and recorded using TriKinetics Drosophila Environment Monitors (DEnM, TriKinetics, Waltham, MA). Temperature-cycling recordings were performed in an MIR-154-PE cooled incubator (Panasonic, Etten-Leur, The Netherlands). All other recordings were performed in SFC3C/RH incubators (LEEC, Nottingham, UK). Both incubator types had computer-controlled LED lighting systems installed (EXLED, Stonehouse, UK).

### (c) Data analysis

Locomotor flight activity was visualized and analysed for daily activity onsets, free-running periods and total activity using ClockLab analysis program version 6 (Actimetrics, Wilmette, IL). ANOVAs and *t*-tests were performed using R version 4.3.1 (R Foundation for Statistical Computing, Vienna, Austria).

Data are presented as actograms showing behavioural activity, and descriptive measures are presented as boxplots depicting median and quartiles, or as violin plots that also depict median and quartiles, with the addition of vertical density indications.

## Data Availability

The manuscript reports behavioural data, of which example actograms are given in the paper. Supplementary material (locomotor recording data) is available online [55].
